# T-regulatory cells exhibit a biphasic response to prolonged endurance exercise in humans

**DOI:** 10.1007/s00421-017-3667-0

**Published:** 2017-06-23

**Authors:** Tom Clifford, Matthew J. Wood, Philip Stocks, Glyn Howatson, Emma J. Stevenson, Catharien M. U. Hilkens

**Affiliations:** 1 School of Biomedical Sciences, Newcastle University, Newcastle upon Tyne, NE1 7RU UK; 2 Musculoskeletal Research Group, Institute of Cellular Medicine, Newcastle University, Newcastle upon Tyne, UK; 3 Department of Sport, Exercise and Rehabilitation, Northumbria University, Newcastle upon Tyne, UK; 4 Water Research Group, School of Environmental Sciences and Development, Northwest University, Potchefstroom, South Africa; 5 Human Nutrition Research Centre, Institute of Cellular Medicine, Newcastle University, Newcastle upon Tyne, UK

**Keywords:** T-regulatory cells, Endurance exercise, Immune dysfunction

## Abstract

**Purpose:**

T-regulatory cells (Tregs) are a sub-population of lymphocytes that act to suppress aberrant immune responses. We investigated changes in the numbers of naïve and terminally differentiated Tregs in the peripheral blood to establish their role in the immuno-suppressive response to prolonged exercise.

**Methods:**

Blood was drawn from seventeen experienced runners (age 40 ± 12 years; height 1.75 ± 0.08 m; mass 71.4 ± 10.8 kg) before, ~1 h after (POST-1h), and on the day following the marathon (POST-1d). Tregs (CD3^+^CD4^+^Foxp3^+^CD25^++^CD127^−^) were analysed in peripheral blood mononuclear cells using flow cytometry. The markers CD45RA and HLA-DR were included to define naïve and terminally differentiated Tregs, respectively.

**Results:**

The absolute number of Tregs decreased (27%) POST-1h marathon (*P* < 0.001) but increased (21%) at POST-1d (*P* < 0.01). Naïve CD45RA^+^ Tregs fell by 39% POST-1h (*P* < 0.01) but were unaffected POST-1d (*P* > 0.05). In contrast, an increased number of Tregs expressing HLA-DR was observed at POST-1d (*P* < 0.01). Interleukin (IL)-1β, IL-6, IL-8 and IL-10 levels in the serum all increased POST-1h (*P* > 0.05) but returned to pre-exercise levels POST-1d. The suppressive cytokine, transforming growth factor-beta, was unaffected by the marathon (*P* > 0.05).

**Conclusions:**

These results suggest that Tregs do not play a major role in immune suppression in the early hours of recovery from a marathon. However, terminally differentiated HLA-DR^+^ Tregs are mobilized the following day, which could represent a compensatory attempt by the host to restore immune homeostasis and limit excessive cell damage.

## Introduction

It is well established that prolonged (>1.5 h), highly strenuous bouts of exercise; a marathon or ultramarathon for example, can transiently perturb immune function, shifting the balance towards a more immunosuppressive state (Gleeson 2007; Nieman 1997, 2007). This immune dysfunction can last for several days after exercise and is typically characterized by a decrease in the total number of circulating lymphocyte cells (Gleeson and Bishop 2005; Handzlik et al. 2013; Nieman et al. 2001), impaired activation and cytolytic function of natural killer (NK) cells (Gleeson and Bishop 2005; Nieman 1997; Nieman et al. 1997), decreased secretion of immunoglobulin A (Gleeson and Pyne 2015) and an altered type 1/type 2 T cell response, in favour of the latter (Martin et al. 2009).

This characteristic shift towards a more immunosuppressive cellular environment after endurance exercise could be driven by changes in the population of circulating T-regulatory cells (Tregs) (Gleeson et al. 2013; Handzlik et al. 2013; Wang et al. 2012; Weinhold et al. 2016). Tregs, which can now be accurately identified by the expression of Fork-head box protein 3 (Foxp3^+^) intracellularly, and CD3^+^CD4^+^CD25^++^CD127^−^ on the cell surface (Weinhold et al. 2016), develop, classically, in the thymus (natural Tregs) but also in the periphery (inducible Tregs), and primarily function to prevent excessive immune responses to self-tissue (Belkaid 2007; Issazadeh-Navikas et al. 2012). Tregs maintain a healthy immune balance by suppressing the function and activity of a broad range of immune effector cells, including CD8^+^ T cells, NK cells, and dendritic cells (Belkaid 2007; Issazadeh-Navikas et al. 2012; Palomares et al. 2010; Sakaguchi et al. 2008). Hence, the proportion of Tregs in the cellular milieu is crucial to maintaining immune homeostasis; if they are produced in excess, they can inhibit the clearance of pathogens, leaving the host more susceptible to new viral or bacterial infections (Belkaid 2007; Liston and Gray 2014). These observations have led to the suggestion that changes in the circulating pool of Tregs might help to explain, at least in part, the increased incidence of upper respiratory tract infections (URTI) reported after prolonged, strenuous exercise (Handzlik et al. 2013; Nieman 1997; Wang et al. 2012).

However, a possible modulatory role for Tregs in acute exercise-induced immunosuppression has only been considered by a limited number of human studies. In addition, the few that have been conducted have come to contrasting conclusions, with some studies suggesting exercise increases the number of circulating Tregs (Wilson et al. 2009; Krüger et al. 2016), decreases circulating Tregs (Perry et al. 2013) or has no effect on circulating Tregs (Handzlik et al. 2013; Rehm et al. 2013). Interestingly, the findings of Perry et al. (2013), that exercise decreases the number of circulating Tregs, runs counter to the supposition that Tregs increase after exercise and function to modulate the immuno-suppressive response (Handzlik et al. 2013; Wang et al. 2012).

In addition to the equivocal findings to date, there are also a number of limitations with these studies that need to be addressed. First, only one of these studies (Rehm et al. 2013) defined Tregs by the expression of CD25^++^/bright cells in conjunction with FoxP3^+^ cells, which is required to accurately distinguish those with high immunosuppressive activity (Fountoulakis et al. 2008). Second, none of the studies to date have measured changes in Tregs in the 3–72 h after exercise, the so-called ‘open window’ period when immune suppression is supposed to be heightened (Nieman 2007). Given these limitations, the primary aim of the present study was to examine peripheral changes in CD3^+^CD4^+^FoxP3^+^CD25^++^CD127^−^ Tregs before, ~1 h, and the day following a marathon. We also examined, for the first time after prolonged, endurance exercise, Tregs expressing CD45RA, to give an indication of the naïve Tregs population (Valmori et al. 2005), and Tregs expressing human leukocyte antigen (HLA)-DR, to give an indication of their differentiation status (Costantino et al. 2008). The changes in circulating Treg cell populations were measured alongside a range of cytokines involved in the immune response to prolonged exercise, and we were especially interested in IL-10 and transforming growth factor-beta (TGF-β), given their relationship with Treg cell function. We hypothesized that Tregs, including those expressing HLA-DR, would increase after the marathon, as would the immunosuppressive cytokines IL-10 and TGF-β.

## Methods

### Participants

Seventeen runners taking part in the Druridge Bay Marathon, Northumberland, UK, volunteered for this investigation. A summary of their physical characteristics and training history is displayed in Table 1. Samples were obtained from a sub-group of participants taking part in a larger study (Clifford et al. 2016); only those whose samples were collected before mid-day were included to limit diurnal variation. The participants completed a health-screening questionnaire to assess the eligibility for the study and were excluded if they had any cardiovascular complications, a recent (<3 months) musculoskeletal injury or were receiving prescribed anti-inflammatory medications other than for asthma. Of the female participants still menstruating, the marathon took place during the early/mid luteal phase. The environmental conditions on the day of the race have been described before (Clifford et al. 2016). Briefly, at the start of the race air temperature was 3.8 °C; humidity 82%; barometric pressure 1013 hpa and wind speed 9 km/h; the weather remained dry throughout and the temp increased to 8.1 °C towards the end of the race. All procedures were carried out as per the guidelines set out in the Declaration of Helsinki. The study received ethical approval from the Faculty of Health and Life Sciences Ethics Committee at Northumbria University. All participants provided written informed consent prior to participation.

**Table 1:** Participants physical characteristics, training history and marathon race data

Physical characteristics	
Age (years)	40 ± 12
Sex (M/F)	12/5
Height (m)	1.75 ± 0.08
Mass (kg)	71.4 ± 10.8
Training history	
No. of years running	11 ± 10
Average training distance (miles/week)	34 ± 11
Longest training run for marathon (miles)	21 ± 2
No. of previous marathons finished	16 ± 20
Marathon race	
Marathon finish time (hh:min:ss)	04:03:00 ± 00:48:09
Pre–post change in body mass (kg)	1.4 ± 1.0
Pre–post plasma volume change	−2.3 ± 4.1

Values are mean ± SD

### Blood sampling

Blood samples were obtained from a branch of the basilica vein at the antecubital fossa on three occasions: the week leading up to the marathon (PRE), ~1 h post-marathon (POST-1h), and the day following the marathon (POST-1d). At all-time points, blood was drawn into a 10 ml vacutainer for serum and 4 ml and 10 ml vacutainers coated with di-potassium ethylene diamine tetra-acetic acid (EDTA). The serum vacutainer was allowed to stand and clot for 30 min before being centrifuged at 3000*g* for 10 min to separate the supernatant. This was subsequently stored in aliquots at −80 °C and only thawed in the morning of the analysis. The 4 ml EDTA vacutainer was transported to a local hospital for haematological analysis. The 10 ml EDTA vacutainer was transported to the Institute of Cellular Medicine, Newcastle University, for peripheral blood mononuclear cell (PBMC) isolation and flow cytometric analysis (see sections below).

### Haematological and cytokine analysis

Haemoglobin, haematocrit, red blood cells (RBC), leukocytes and lymphocyte counts were measured in whole blood using an automated haematology system (Sysmex XE-2100, IL, USA). According to data provided by the laboratory, the coefficient of variation (CV) for these procedures are typically <10%. Haemoglobin and haematocrit were used to calculate pre–post marathon changes in plasma volume according to the equations of Dill and Costill (1974). Interleukin (IL)-1ra, Il-1β, IL-2, IL-4, IL-6, IL-8, IL-10, TNF-α and IFN-γ were measured in serum as part of a multiplex sandwich chemiluminescent immunoassay kit (Evidence Investigator, Randox Laboratories, Northern Ireland, UK). Inter and intra assay CVs for this analysis was <5%. TGF-β was analysed in serum using a commercially available ELISA kit (R&D Systems, Minneapolis, USA). Intra-assay CV for this analysis was <15%.

### Peripheral blood mononuclear cell isolation

Peripheral blood mononuclear cells were purified by density gradient centrifugation. Blood samples collected from 10 ml EDTA vacutainers were added to a leucosep tubes (Grenier bio-one) and centrifuged for 10 min at 900*g*. PBMCs were recovered from the interphase and washed at 600 and 250*g* for 7 min. Cell washes were carried out with HBSS cell wash media (Hanks Balanced Salt Solution (Lonza) supplemented with 10% fetal bovine serum (FBS) (Life Technologies BRL), and cell pellet re-suspended in appropriate medium unless stated otherwise.

### Cryopreservation and thawing of cells

Peripheral blood mononuclear cell pellets were first re-suspended in FBS, and then an equal volume of FBS +20% DMSO (Sigma-Aldrich) was added dropwise to the cell suspensions (final concentration – FBS + 10% DMSO). PBMC suspensions were split between two cryovials, then placed in a room temperature cool cell and stored at −80 °C. This ensures freezing occurs at a rate less than 1 °C/min. For thawing, cryovials were placed in a 37 °C water bath for 10 min. The PBMC suspension was then transferred to a 30 ml universal tube, and 10 ml of pre-warmed thawing medium (RPMI-1640 + supplemented with 10% FBS) was added slowly, at a rate of <1 ml/5 s. The cells were washed twice at room temperature at 400*g* for 7 min, and resuspended in PBS.

### Flow cytometry analysis

**Fig. 1:**
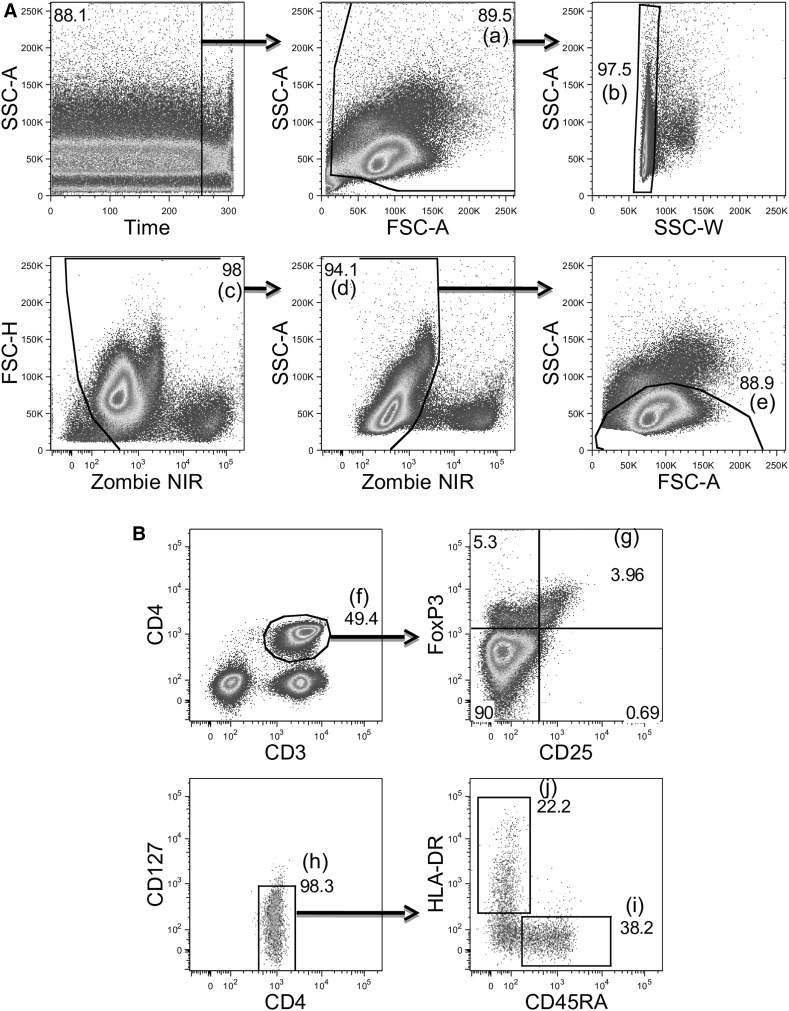
Gating strategy to define Treg subsets. **A** Gating was performed to exclude debris (*a*), doublets (*b*), red blood cells (*c*) and dead cells (*d*) using time during acquisition, forward and side scatter properties and viability dye zombie NIR. Final gating is on the lymphocyte population (*e*). **B** Gating strategy to define Treg subsets within the CD3^+^CD4^+^ population (*f*): CD25^++^Foxp3^+^ (*g*) and CD127^−^ total Tregs (*h*); CD45RA^+^ Tregs (*i*) and HLA-DR^+^ Tregs (*j*)

Flow cytometry analysis is expressed as changes in the  % of total lymphocytes and the absolute number of cells per μl of peripheral blood. Total Tregs and Treg cell subpopulations (‘naïve’ CD45RA^+^ and ‘mature’ HLA-DR^+^) are also expressed as a % of total CD3^+^CD4^+^ cells. The gating strategy used to define CD3^+^CD4^+^ cells and Tregs is displayed in Fig. 1. Briefly, in a sequential fashion, gating was performed to exclude debris; followed by gating on the total lymphocyte population in the FSC/SSC plot. Lymphocytes co-expressing CD3^+^CD4^+^ were then gated on to determine the % of CD3^+^CD4^+^ cells in the total lymphocyte population. For identification of Tregs, the CD3^+^CD4^+^ cell population was gated on by initially plotting CD25 against FoxP3 to determine the CD25^++^FoxP3^+^ population, followed by gating on the CD127^−^ population, giving CD3^+^CD4^+^Foxp3^+^CD25^++^CD127^−^ Tregs. Tregs were subsequently gated for CD45RA and HLA-DR expression. Cells expressed as a % of total lymphocytes and % of total CD3^+^CD4^+^ cells were calculated by the FlowJo analysis software following the above gating strategy.

### T-regulatory cell analysis

Thawed PBMC were stained with viability dye Zombie NIR (Biolegend) at room temperature for 15 min. Cells were washed, resuspended in blocking buffer [PBS + 9% human IgG (Sigma, Poole, UK) + 27% mouse serum (Sigma)] and stained with following anti-human monoclonal antibody cocktail: anti-CD3-Alexa Fluor 488 (UCHT1); anti-CD4-V500 (RPA-T4); anti-CD25-APC (M-A251); anti-CD45RA-Alexa Fluor 700 (HI100); anti-CD127-PE-Cy7 (HIL-7R-M2); anti-HLA-DR-V450 (L243) (all BD Biosciences, CA, USA). After incubation at 4 °C for 20 min, cells were fixed and permeabilized using appropriate buffers (FoxP3 Fixation/Permeabilization Concentrate and diluent kit; eBioscience Ltd, Hatfield, UK) then resuspended in perm/wash buffer (BD Biosciences). Intracellular staining of Fox-P3 was achieved using anti-FoxP3-PE (236A/E7, BD Biosciences). Cells were incubated at 4 °C for 30 min then washed and resuspended in fluorescence-activated cell sorting (FACS) buffer (PBS + 0.5% BSA + 0.01% sodium azide). Data were acquired on a LSR Fortessa X20 (BD Biosciences) and analysed using FlowJo Version 8.8.7 (Treestar, Ashland, OR, USA).

### Data analysis

All data were analysed using GraphPad Prism (GraphPad Software Inc., CA, USA) and expressed as mean ± SD unless stated otherwise. Paired *t* tests were used to analyse pre–post marathon changes in mass and plasma volume (PV). Time course changes from PRE to POST-1h or POST-1d for cytokine, haematological and Treg cell variables were adjusted for PV changes according to the methods of Dill and Costill (1974) and subsequently analysed using a one-way analysis of variance (ANOVA), with Bonferroni corrections applied for multiple comparisons. Cohen’s *d* effect sizes (ES) were calculated with the magnitude of effects considered small (0.2–0.49), medium (0.5–0.79) and large (≥0.8). Statistical significance was set at *P* < 0.05 prior to analyses.

## Results

### Haematology

Changes in haematological parameters induced by the marathon are presented in Table 2. Total blood lymphocyte concentrations dropped by 20% POST-1h (*P* = 0.02; ES = 0.72) but had returned to baseline by POST-1d (*P* > 0.05; ES = 0.46). Whole blood leukocyte concentrations were 160 and 21% higher than PRE values at POST-1h and POST-1d, respectively (*P* < 0.01). Both neutrophils and monocytes were above PRE concentrations at POST-1h and POST-1d (*P* < 0.001). Eosinophils fell by 68% POST-1h (*P* < 0.01; ES = 1.24) but returned to PRE values by POST-1d (*P* > 0.05; ES = 0.17). Basophil counts were elevated above PRE at POST-1h (*P* = 0.02; ES = 0.43) but not significantly at POST-1d (*P* > 0.05). In summary, while lymphocytes and eosinophils fell below baseline levels after the marathon, phagocytic leukocytes (neutrophils and monocytes) were elevated post-marathon and remained elevated the following day.

**Table 2:** Haematology before and after the marathon

Biomarker	PRE	POST-1h	POST-1d
Lymphocytes (10^9^ cells/l)	1.99 ± 0.54	1.61 ± 0.50*	2.26 ± 0.61
Leukocytes (10^9^ cells/l)	5.5 ± 1.1	14.3 ± 2.8*	6.6 ± 1.0*
Neutrophils (10^9^ cells/l)	2.8 ± 0.8	11.5 ± 2.6*	3.6 ± 1.2*
Monocytes (10^9^ cells/l)	0.5 ± 0.1	1.0 ± 0.4*	0.6 ± 0.2*
Eosinophils (10^9^ cells/l)	0.19 ± 0.13	0.06 ± 0.07*	0.16 ± 0.11
Basophils (10^9^ cells/l)	0.03 ± 0.01	0.04 ± 0.02*	0.04 ± 0.01

One-way ANOVA was used to test for time differences

*PRE* before the marathon, *POST-1h* 1 h after the marathon, *POST-1d* 1 day after the marathon

* Different from pre-race (*P* < 0.05)

### Cytokines and growth factors

A number of cytokines and growth factors were measured to determine their role in the inflammatory response after the marathon, and if changes in these parameters were associated with changes in Treg cell activity; these results are displayed in Table 3. At POST-1h, there was a large increase in IL-6 (3062%; ES = 2.96), IL-8 (136%; ES = 1.48) and IL-10 (2022%; ES = 2.14) (*P* < 0.01), and a moderate increase in TNF-α (15%; ES = 0.39) and IL-1β (62%; ES = 0.87) (*P* < 0.05). All cytokines had returned to PRE levels by POST-1d (*P* > 0.05). IL-1ra, IL-1, IFN-γ, IL-2, IL-4, TGF-β did not rise appreciably above PRE at any time point after the marathon (*P* > 0.05). Therefore, the marathon stimulated only a transient cytokine response that was not evident the day after the marathon, and thus, these cytokines did not seem to be associated with the elevated Treg cell activity at this time point (see below).

**Table 3:** Cytokine levels is serum before and after the marathon

Biomarker (pg/ml)	PRE	POST-1h	POST-1d
IL-1ra	0.43 ± 0.34	0.37 ± 0.23	0.35 ± 20
Il-1β	1.64 ± 0.99	2.66 ± 1.32*	1.86 ± 0.79
IL-2	3.9 ± 4.0	5.9 ± 8.8	3.8 ± 3.6
IL-4	2.2 ± 0.6	2.1 ± 0.5	2.4 ± 0.7
IL-6	1.0 ± 0.5	33.1 ± 21.1*	1.7 ± 1.2
IL-8	8.7 ± 5.7	20.6 ± 10.3*	7.9 ± 5.0
IL-10	1.1 ± 0.5	23.5 ± 20.4*	0.9 ± 0.2
TNF-α	2.9 ± 1.1	3.4 ± 1.1*	2.9 ± 1.1
TGF-β	19010 ± 9876	20907 ± 8991	17876 ± 9479
IFN-γ	0.55 ± 0.70	0.44 ± 0.62	0.46 ± 0.58

*PRE* before the marathon, *POST-1h* 1 h after the marathon, *POST-1d* 1 day after the marathon, *IL* interleukin, *ra* receptor antagonist, *TNF-α* tumour necrosis factor-alpha, *TGF-β* transforming growth factor-beta, *IFN-γ* interferon-gamma

* Different from pre-race (*P* < 0.05)

### CD3^+^CD4^+^ lymphocytes

**Fig. 2:**
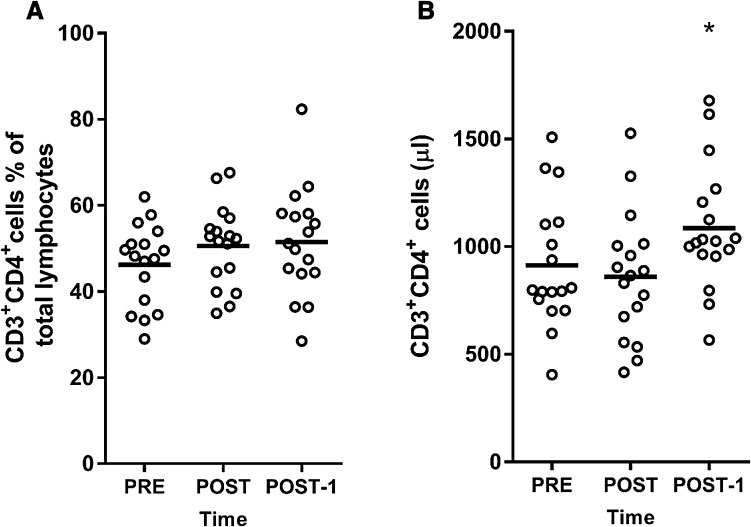
CD3^+^CD4^+^ cells PRE (before the marathon); POST-1h (1 h after the marathon); POST-1d (1 day after the marathon). **a** Percentage of CD3^+^CD4^+^ cells in total lymphocytes, **b** total CD3^+^CD4^+^ cells (number per µl in blood). *Asterisk* denotes higher than PRE (*P* < 0.05); one-way ANOVA was used to test for time differences

Cells expressing CD3^+^CD4^+^ were measured to give an indication of overall T helper-cell mobilization within the total lymphocyte population. The % of CD3^+^CD4^+^ cells in the total lymphocyte pool was not significantly increased at POST-1h or POST-1d (*P* > 0.05; Fig. 2a). In contrast, the absolute numbers of CD3^+^CD4^+^ cells were 14% higher at POST-1d (*P* = 0.01; ES = 0.59) but unchanged at POST-1h compared to PRE (*P* > 0.05; ES = 0.18; Fig. 2b). Thus, while the absolute number of circulating CD3^+^CD4^+^ increased the day after, they were unchanged in the immediate hours following the marathon.

### Treg cell subpopulations

**Fig. 3:**
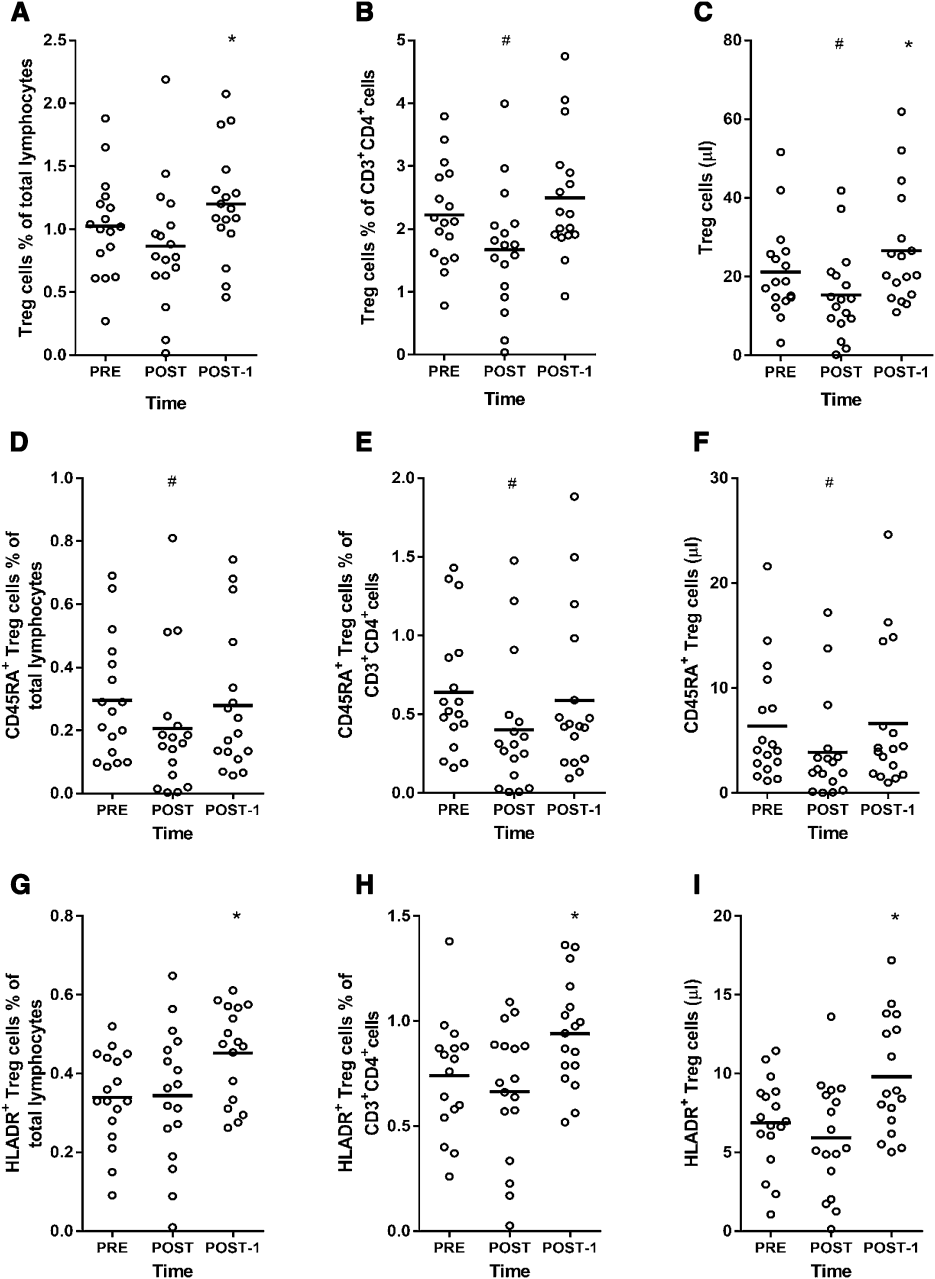
T-regulatory (Treg) cells PRE (before the marathon); POST-1h (1 h after the marathon); POST-1d (1 day after the marathon). **a** Percentage of Tregs in total lymphocytes, **b** percentage of Tregs in CD3^+^CD4^+^ cells, **c** total Tregs (number per µl in blood), **d** percentage of CD45RA^+^ Tregs in total lymphocytes, **e** percentage of CD45RA^+^ Tregs in CD3^+^CD4^+^ cells, **f** total CD45RA^+^ Tregs (number per µl in blood), **g** percentage of HLADR^+^ Tregs in total lymphocytes, **h** percentage of HLADR^+^ Tregs in CD3^+^CD4^+^ cells, **i** total HLADR^+^ Tregs (number per µl in blood). *Hash symbol* denotes lower than PRE (*P* < 0.05); *asterisk* denotes higher than PRE (*P* < 0.05). One-way ANOVA was used to test for time differences

Because Tregs are believed to play an important role in immunosuppression, and thus, might heighten UTRI risk after long duration exercise, we analysed peripheral changes in these cells up to 1 day following the marathon. Changes in the population of all Tregs subsets analysed are presented in Fig. 3. The % of total Tregs (CD3^+^CD4^+^FoxP3^+^CD25^++^CD127^−^) in total lymphocytes did not change POST-1h (*P* = 0.08; ES = 0.35) but increased at POST-1d (*P* = 0.02; ES = 0.42; Fig. 3a). There was a decrease in the % of Tregs in CD4^+^ cells POST-1h (*P* = 0.01; ES = 0.62) but no change from PRE at POST-1d (*P* = 0.13; ES = 0.30; Fig. 3b). The mean number of absolute Tregs at PRE was 21.1 ± 11.8 (cells/µl). At POST, cell numbers decreased to 15.3 ± 11.3 (*P* = 0.01; ES = 0.50); but at POST-1 increased to 26.6 ± 14.7 (*P* = 0.01; ES = 0.41; Fig. 3c). Thus, the number of Tregs decreased ~1 h after the marathon before rebounding, and increasing above pre-exercise values the following day.

Tregs expressing CD45RA^+^ were analysed to characterize changes in the naive Treg cell population induced by the marathon. The % of CD45RA^+^ Tregs in total lymphocytes (Fig. 3d) and % in CD4^+^ cells (Fig. 3e) decreased at POST-1h (*P* = 0.01; ES = 0.43 and *P* = 0.01; ES = 0.57, respectively) but were not different to PRE levels by POST-1d (*P* > 0.05). A similar pattern was observed for absolute cell counts of CD45RA^+^ Tregs, which decreased by 39% POST-1h (*P* = 0.01; ES = 0.48) but were similar to PRE at POST-1d (*P* > 0.05; ES = 0.03; Fig. 3f). These data indicate that the naïve Treg cell population decreased in the immediate hours after the marathon but was restored to pre-exercise levels by the following day.

We analysed Tregs cells expressing the HLA-DR marker, which is purported to represent a distinct and mature population of Tregs, displaying enhanced suppressive activity and mainly acting in a cell contact-dependent manner (Costantino et al. 2008). We found that the % of HLA-DR^+^ Tregs in total lymphocytes was unchanged at POST-1h (*P* > 0.05; ES = 0.03) but increased from 0.33 ± 0.11 to 0.45 ± 0.11 at POST-1 (*P* = 0.01; ES = 0.95; Fig. 3g). The proportion of HLA-DR^+^ Tregs within the CD3^+^CD4^+^ population were also higher than PRE at POST-1d (*P* = 0.01; ES = 0.75; Fig. 3h). Similarly, the mean number of absolute HLA-DR^+^ Tregs was unchanged POST-1h (*P* > 0.05; ES = 0.29) but increased by 42% at POST-1d (*P* = 0.01; ES = 0.88; Fig. 3i). Therefore, the number of mature, terminally differentiated HLA-DR^+^ Tregs, remained unchanged in the immediate hours post-marathon, but were significantly and substantially increased the day following the marathon.

## Discussion

In the present study, we evaluated changes in circulating Tregs before and up to 1 day following a marathon. We found that: (1) Tregs exhibited a biphasic response, whereby post-marathon, they decreased, but the following day, they rebounded, and increased above pre-marathon levels, and; (2) the number of HLA-DR^+^ and, therefore, mature Tregs, significantly increased the day after the marathon.

The finding that Tregs decreased below pre-marathon levels ~1 h post are in agreement with those of Perry et al. (2013), who also found that Tregs decreased after a marathon or ironman triathlon race. Yet they are in contrast to those of Wilson et al. (2009) and Krüger et al. (2016) who reported that Tregs increased after high intensity interval swimming and cycling exercise, respectively. Others found no changes in Treg cell numbers after 30 or 60 min of sub-maximal cycling exercise (Handzlik et al. 2013; Krüger et al. 2016). The discrepancy in findings between these studies and ours could simply be due to the different methods used to quantify and define Tregs. However, another possible explanation is related to the different types and duration of exercise. In ours and the study of Perry et al. (2013), in which the exercise stimulus was much longer in duration (≥3 h), Tregs decreased after exercise, whereas in the studies that were short duration (≤30 min), but performed at a high intensity, Tregs increased (Krüger et al. 2016; Wilson et al. 2009). In contrast, studies in which the exercise stimulus was of a moderate intensity (~70% *V*O_2max_) had no effect on circulating Treg cell numbers (Handzlik et al. 2013; Krüger et al. 2016). Taken together, it seems that Tregs do not play a major role in suppressing the immune system after moderate intensity exercise, or prolonged endurance exercise—at least in the early stages of recovery—but may after short duration, high intensity exercise.

A possible explanation for the decrease in Tregs is that the marathon stimulated cell death via apoptosis. Although Treg cell death was not measured in this study, a recent study (Krüger et al. 2016) found that continuous aerobic exercise (30 min of cycling at 70% *V*O_2max_) elicits apoptosis in peripheral Tregs, which lends some support to this idea. Exercise-induced Treg cell apoptosis would also be wholly consistent with the effects of aerobic exercise on other T-cell sub-populations (Krüger et al. 2016; Navalta et al. 2013). Alternatively, it is also important to consider that the decrease in circulating Tregs simply reflects the fact that they were rapidly distributed to other tissues after the marathon, as suggested with other T-cells populations (Krüger et al. 2016; Krüger et al. 2008). Further research is needed to determine the biochemical processes to explain the decrease in Tregs after the marathon and also the clinical significance of this finding.

The day after the marathon, there was a significant increase in CD3^+^CD4^+^ cells, which could represent mobilization of the T-cell population (Gleeson and Bishop 2005; Walsh et al. 2011). Perhaps of most importance, there was a parallel increase in the number of Tregs expressing HLA-DR, representing terminally differentiated, mature, Tregs. The fact that the proportion of HLA-DR^+^ Tregs in the CD3^+^CD4^+^ and total lymphocyte populations also increased suggests that these cells might have been preferentially expanded/mobilized within the whole T-cell pool. Importantly, these findings not only provide the first evidence that Treg cell numbers are increased the day after a marathon, but that in particular the number of mature Tregs, known to have enhanced suppressive activity, have gone up. The mechanism by which HLA-DR^+^ Tregs are preferentially mobilised 1 day after a marathon remains to be investigated.

The reason for the delayed Treg cell response after the marathon is unclear, but since the main function of Tregs is to terminate T-cell effector responses and, broadly, general suppression of the immune system (Issazadeh-Navikas et al. 2012), it could be that this response signifies a compensatory attempt to limit excessive cell damage, possibly that caused during the reconstruction of dysfunctional cells. Indeed, it is well established that strenuous physical exercise stimulates a sequential process of inflammation and regeneration that first involves the destruction of damaged or necrotic cells (Chazaud 2016; Tidball and Villalta 2010). It would be reasonable to assume that the accumulation of Tregs in the circulation was in response to such effects, and that perhaps these cells were being mobilized for distribution to various tissues for reparative processes, the most obvious being skeletal muscle. Burzyn et al. (2013) found that Tregs accumulate in damaged skeletal muscle of mice, lending some support to this idea. The authors proposed that their role might be in orchestrating the characteristic switch from a pro-inflammatory to anti-inflammatory environment, which promotes satellite cell proliferation and muscle healing. This suggests that Tregs increased the day after the marathon to restore normal immune function.

Post-marathon, there was a decrease in the absolute number of CD45RA^+^ Tregs and their proportion in the total lymphocyte and CD3^+^CD4^+^ populations. This is the first study to measure Tregs expressing CD45RA after a marathon, but these findings are consistent with a previous study that, although they did not specifically define CD45RA^+^ Tregs, they did find CD4^+^ CD45RA^+^ cells (and thus presumably the whole T-cell pool) to increase after high intensity running exercise (Simpson et al. 2007). The significance of our finding is unclear, and it is possible that the decrease in Tregs expressing CD45RA is just a reflection of the decrease in the total Treg cell population. Alternatively, given that the expression of CD45RA cells is believed to represent an immunologically naïve population of T-cells, it is likely that the decrease in CD45RA^+^ signifies a switch from naïve Tregs to memory/activated Tregs expressing the CD45RO variant (Seddiki et al. 2006).

The leukocyte response followed a similar pattern to that reported for previous marathon studies (Nieman et al. 2001; Shanely et al. 2014; Suzuki et al. 2000). The increase in total leukocytes, especially the neutrophilia and monocytosis has consistently been observed after marathons (Nieman et al. 2001; Shanely et al. 2014; Suzuki et al. 2000) and is probably due to the secretion of cytokines, chemokines and stress hormones during and in the immediate hours after the race (Slattery et al. 2015; Nieman et al. 2001). The continued elevation in neutrophils and monocytes the following day might be a response to any cardiac or skeletal muscle damage resulting from the marathon (Hikida et al. 1983; Paulsen et al. 2010; Whyte 2008).

As in the majority of previous studies, we observed a significant increase in IL-6, IL-8 and IL-10, after the marathon (Shanely et al. 2014; Suzuki et al. 2000, 2003), which further confirms the important modulatory role of these cytokines in the early immune response to endurance exercise. Interestingly, none of the cytokines measured in this study were upregulated the day after the marathon. In this regard, the most surprising finding was that TGF-β, a suppressive cytokine produced by Tregs, remained unchanged after the marathon. Although not a consistent finding (Suzuki et al. 2000), two studies reported increased TGF- β in response to a marathon (Perry et al. 2013; Toft et al. 2000) and, given its relationship with Tregs, we anticipated TGF-β and Tregs to follow a similar pattern of change after the marathon. Exactly why TGF-β was unchanged in the present study, but was in others (Perry et al. 2013; Toft et al. 2000) is unclear. Differences in study design, including the timing of measurements, exercise intensity, participants, and analytical procedures are all feasible explanations. It is also possible that had we analysed just active TGF-β, as opposed to total TGF-β (therefore, excluding latent TGF- β), then we would have seen increases above baseline following the marathon. Nonetheless, our findings suggest that the increase in mature HLA-DR^+^ Tregs was largely independent of total TGF-β (and IL-10) production after the marathon, which is in keeping with this Treg population primarily inducing its suppressive effects via cell-contact dependent mechanisms (Baecher-Allan et al. 2006; Costantino et al. 2008).

It is important to acknowledge the limitations of this study. First, as our study was performed in endurance trained athletes, these findings are unlikely to be generalizable to exercise naïve individuals or even athletes unaccustomed to endurance exercise. Another important limitation of this study is that our analysis was limited to the circulation, and thus, we cannot exclude the possibility that Treg cell activation displays a different pattern of change in other tissues after endurance exercise. The biological relevance of the changes observed in this study also requires investigation.

In conclusion, our results show that the Treg cell response to a marathon is biphasic; after initially decreasing in the early stages of recovery, they increase the following day, presumably to limit excessive cell damage. These findings suggest that, contrary to recent suggestions (Handzlik et al. 2013; Wang et al. 2012; Weinhold et al. 2016), Tregs do not appear to be major contributors to the immediate immuno-suppressive effect of prolonged, strenuous exercise. Additionally, they also suggest that Tregs might play a key role in limiting cell dysfunction arising from exercise-related inflammation and perhaps they could be manipulated (e.g. via dietary changes; Issazadeh-Navikas et al. 2012) to promote the restoration of cell homeostasis after exercise.

## Abbreviations

Anova: Analysis of variance
CV: Coefficient of variation
DMSO: Dimethyl sulfoxide
EGF: Epidermal growth factor
FACS: Fluorescence activated cell sorter
FBS: Fetal bovine serum
Foxp3: Fork-head protein 3
HLA-DR: Human leukocyte antigen-D related
NK: Natural killer
IL-1-ra: Interleukin 1-receptor agonist
IL-1β: Interleukin-1beta
IL-2: Interluekin-2
IL-4: Interleukin-4
IL-6: Interleukin-6
IL-8: Interleukin-8
IL-10: Interleukin-10
INF-γ: Interferon-gamma
MCP-1: Monocyte chemoattractant protein 1
PBMC: Peripheral blood mononuclear cell
RBC: Red blood cells
RONS: Reactive oxygen and nitrogen species
TGF-β: Transforming growth factor-beta
TNF-α: Tumour necrosis factor-alpha
Tregs: T-regulatory cells
URTI: Upper respiratory tract infection
VEGF: Vascular endothelial growth factor
